# Inter-Species Host Gene Expression Differences in Response to Human and Avian Influenza A Virus Strains

**DOI:** 10.3390/ijms18112295

**Published:** 2017-11-01

**Authors:** Biruhalem Taye, Dawn Yeo, Raphael Tze Chuen Lee, Boon Huan Tan, Richard J. Sugrue, Sebastian Maurer-Stroh

**Affiliations:** 1 School of Biological Sciences, Nanyang Technological University, 60 Nanyang Drive, Singapore 637551, Singapore; biruhalem@bii.a-star.edu.sg (B.T.); ysuyin@dso.org.sg (D.Y.); 2 Bioinformatics Institute (BII), 30 Biopolis Street, #07-01 Matrix Building, Singapore 138671, Singapore; leetc@bii.a-star.edu.sg; 3 Aklilu Lemma Institute of Pathobiology, Addis Ababa University, Addis Ababa P.O. Box 1176, Ethiopia; 4 Detection and Diagnostics Laboratory, DSO National Laboratories, 27 Medical Drive, Singapore 117510, Singapore; tboonhua@dso.org.sg; 5 LKC School of Medicine, Nanyang Technological University, 60 Nanyang Drive, Singapore 637551, Singapore; 6 Department of Biological Sciences (DBS), National University Singapore (NUS), 14 Science Drive 4, Singapore 117543, Singapore

**Keywords:** influenza A virus, inter-species transcriptome, apoptosis, metabolic pathways

## Abstract

Low pathogenic avian influenza (LPAI) viruses are a source of sporadic human infections and could also contribute to future pandemic outbreaks but little is known about inter-species differences in the host responses to these viruses. Here, we studied host gene expression signatures of cell lines from three species (human, chicken, and canine) in response to six different viruses (H1N1/WSN, H5N2/F59, H5N2/F118, H5N2/F189, H5N3 and H9N2). Comprehensive microarray probe set re-annotation and ortholog mapping of the host genes was necessary to allow comparison over extended functionally annotated gene sets and orthologous pathways. The annotations are made available to the community for commonly used microarray chips. We observe a strong tendency of the response being cell type- rather than virus-specific. In chicken cells, we found up-regulation of host factors inducing virus infectivity (e.g., oxysterol binding protein like 1A (*OSBPL1A*) and Rho GTPase activating protein 21 (*ARHGAP21*)) while reducing apoptosis (e.g., mitochondrial ribosomal protein S27 (*MRPS27*)) and increasing cell proliferation (e.g., COP9 signalosome subunit 2 (*COPS2*)). On the other hand, increased antiviral, pro-apoptotic and inflammatory signatures have been identified in human cells while cell cycle and metabolic pathways were down-regulated. This signature describes how low pathogenic avian influenza (LPAI) viruses are being tolerated and shed from chicken but potentially causing cellular disruption in mammalian cells.

## 1. Introduction

Influenza A virus (IAV) is a zoonotic virus that is present in several animal reservoirs (e.g., avian, pigs) [1]. These animal viruses play an important role in the evolution of influenza viruses that circulate with the human population. However, several barriers to infection must be overcome when viruses are transmitted from an avian to a human host. Furthermore, several additional obstacles must be overcome for the virus to be maintained in the new host [1]. In essence, the virus must adapt to overcome these barriers to infection. Although there have been extensive studies to understand the basis for the host adaptations that allow maintenance of the avian influenza virus in the new mammalian host, this process it is currently not fully understood [2]. However, a combination of high mutation rates and gene reassortment is a major driver in mediating host adaptation [3].

Several IAV proteins have been implicated in mediating host adaptation mutations. Human and avian influenza viruses primarily infect ciliated cells that express α (2, 6-linked) sialic acid receptor and α (2, 3-linked) sialic acid receptor respectively [4], and mutations in hemagglutinin (HA) protein can allow avian viruses to adapt to infect mammals [5]. In addition, several host range determinants are located in the polymerase proteins, which is important for the maintenance of the virus in the new host. For example the avian virus (H5N1) polymerases have been demonstrated to have poor activity in mammalian hosts (e.g., human), and acquiring of specific mutations in the polymerase complex proteins (polymerase basic protein 1 (PB1), polymerase basic protein 2 (PB2) and polymerase acidic protein (PA)), the nucleoprotein (NP) and the nuclear export protein (NEP) is associated with mammalian host adaptation [6,7].

Although several studies have focused on the identification of virus gene sequence variation that determine species adaptation, the host responses that are elicited by the different viruses is also important in the context of species adaptation. In particular, the specific effects that virus infection has on general cell physiology would be expected to be an important factor in enabling a virus to replicate in a new host. Although animal models of infection can provide useful in vivo information about pathogen host interactions, the influenza viruses usually need to be adapted to the specific animal model that is used [8]. This process of adaptation that is often required to replicate in a non-natural host (e.g., mice) can change fundamental properties of the virus isolates, which are both multi-factorial and poorly defined. In addition, if a virus is not able to replicate efficiently in the animal model this may also impact on other biological properties of the virus that requires efficient virus replication e.g., such as the magnitude of the host response. In this context, cell types that are normally permissive to influenza virus replication represent a convenient model system to examine influenza virus replication. In these systems the virus infection can be accurately controlled, allowing the identification of virus-induced cellular changes that occur early in infection.

Human alveolar basal epithelial (A549), Madin-Darby canine kidney (MDCK), and Chick embryo fibroblasts (CEF) cells represent the most common cell types that are used to examine influenza virus infection. In addition, MDCK cells are highly permissive to IAV infection allowing propagation of clinical and veterinary strains of influenza virus. Although these cell types would be expected to exhibit transcription profiles that are distinct from the tissue they are derived, it would be expected that signaling networks (e.g., such as those related to the innate host response to infection) that are present within these cells types would retain species-specific properties (e.g., how they are regulated). In addition, since these cells are highly permissive for influenza virus infection they would serve as good models to understand the cellular changes that IAVs induce that are pro-viral in nature. We have previously described several low pathogenic avian influenza (LPAI) viruses H5N2, H5N3 and H9N2 that were isolated from live ducks that were imported into Singapore [9]. The replication characteristics of these viruses in A549, MDCK and CEF cells were examined in detail [10], and interestingly, the avian viruses exhibited a cell-specific capacity to replicate in these cell types. We can hypothesize that the capacity of specific virus isolates to modulate to host cell transcriptome may determine the capacity of viruses to replicate in mammalian and avian cell types, and by extrapolation, different animal hosts. However, the annotations of probe sets in the chicken and canine genome which are less well annotated compared to those from human, with 50% and 40% of the chicken and canine probe sets respectively being not functionally annotated [11]. Incomplete annotation of microarray probe sets prevented a detailed side-by-side comparison of the host responses in these different cell types, and limited the interpretation of expression results in the different cell types. In this current study we have extended our previous analysis by applying bioinformatics tools to annotate and find ortholog mappings between the different species which has enabled us to compare the virus-specific expression signatures in the different cell types. This approach is able to highlight the key expression signature differences between different species during IAV infections and gives insight to the host-response mechanism against IAVs.

## 2. Results

### 2.1. Annotation and Orthologs Mapping

**Figure 1:**
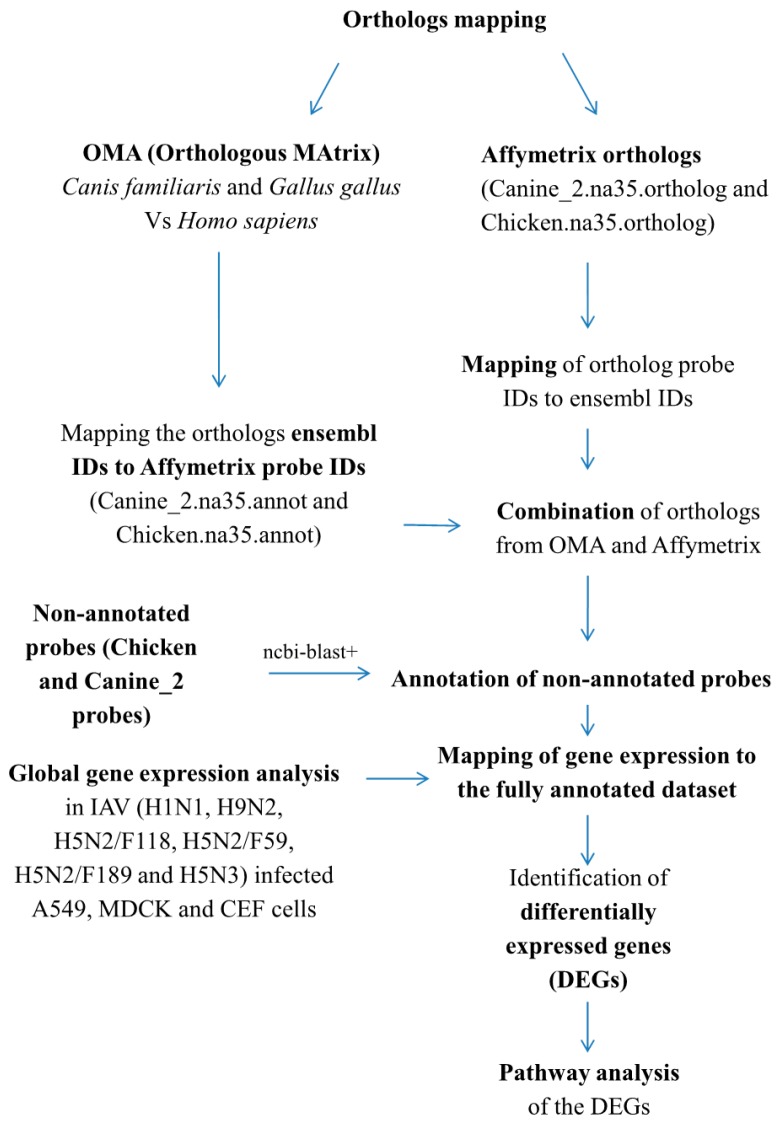
Overview of Affymetrix probe annotation and orthologs mapping. IDs = identifications, OMA = “Orthologous Matrix” database.

In this study the GeneChip Canine Genome 2.0 Array (Affymetrix, Santa Clara, CA, USA), the GeneChip Chicken Genome Array (Affymetrix) and Genechip Human Genome HG U133 Plus 2.0 Array (Affymetrix) probe sets were used for annotations, orthologs mapping and gene expression analysis (Figure 1). First we mapped the non-annotated probe sets to Ensembl reference gene sets of chicken and canine genomes as basis for probe annotation. We observed that 41.6% (16,055/38,535) and 30.6% (13,195/43,035) of the chicken genome and canine genome Affymetrix arrays were not annotated with ensembl identifications (IDs) (Table 1). Using ncbi-blast+ package we are able to annotate 99.52% (15,979/16,055) and 99.7% (13,162/13,195) of the non-annotated probes with ensembl IDs in chicken and canine genome arrays, respectively (Table 1).

The annotated probes represent 7412 chicken and 6869 canine genes. Although 84% (6229/7412) and 85.9% (5899/6869) of the annotated chicken and canine genes respectively are represented by other Affymetrix annotated probes, still nearly 52.9% (7412/14,007) chicken and 38.5% (6869/17,813) canine genes would have been missed if the probes were not annotated. In addition, we used annotations from ensembl genes 83, NCBI and DAVID for annotation of ensembl IDs/probes to official gene symbols and with the support of orthologs mapping (see below), we increased the annotation of the chicken and canine genome arrays from 58.4% to 99.8 % (38,474/38,535) and 69.4% to 99.9% (43,006/43,035), respectively (Table 1 and Table S1).

As indicated in Table 2, only 35–37% of the chicken and canine genome arrays were mapped to human orthologs (human genome HG U133 Plus 2.0 array). To maximize the coverage, we analyzed human orthologs of chicken and canine genes using the “Orthologous Matrix” (OMA) database [12]. Overall, 11,674 and 16,002 chicken and canine genes respectively are orthologs to human genes (Table 2 and Table S1). In chicken, 11,220 of the 11,674 genes were common to the fully annotated probes-ensembl genes and represent 80% of the array genes. Similarly, 15,284 of the 16,002 human orthologs of the canine genes were in the fully annotated sets representing 85.8% of the canine genome array. After mapping probes to ensembl IDs and vice versa, the combination of orthologs from OMA and Affymetrix covered 89.1% and 91.6% of the chicken and canine genome array probes, respectively (Table 2 and Table S1).

### 2.2. Comparison of Differentially Expressed Genes (DEGs) in A549 with Madin-Darby Canine Kidney (MDCK) and Chick Embryo Fibroblasts (CEF) Cells

The microarray expression data was analyzed using GEO2R (limma, R-package, Bioconductor, NY, USA) and the comparison of differentially expressed genes (DEGs) in chicken (CEF) and canine (MDCK) relative to their human orthologs (A549) is described in Table 3 and Table 4. Generally, the expression changes were mainly observed after 6 hpi. The numbers of DEGs were significantly higher in MDCK than A549 cells, particularly at 10 hpi. This is similar to the number of down-regulated genes except in H5N3; however the reverse is true for the up-regulated genes (Table 3). Unlike A549 and MDCK cells, the numbers of both up and down-regulated genes were significantly higher in A549 cells compared to CEF with exception of H5N2/F118 (Table 4). Taken together, the data suggested the expression changes in IAV infection are in the order of MDCK > A549 > CEF with exceptions of up-regulated genes in A549 vs. MDCK and the H5N2/F118 virus infection in A549 vs. CEF cells.

### 2.3. Functional Annotation of the DEGs at 10 hpi

**Figure 2:**
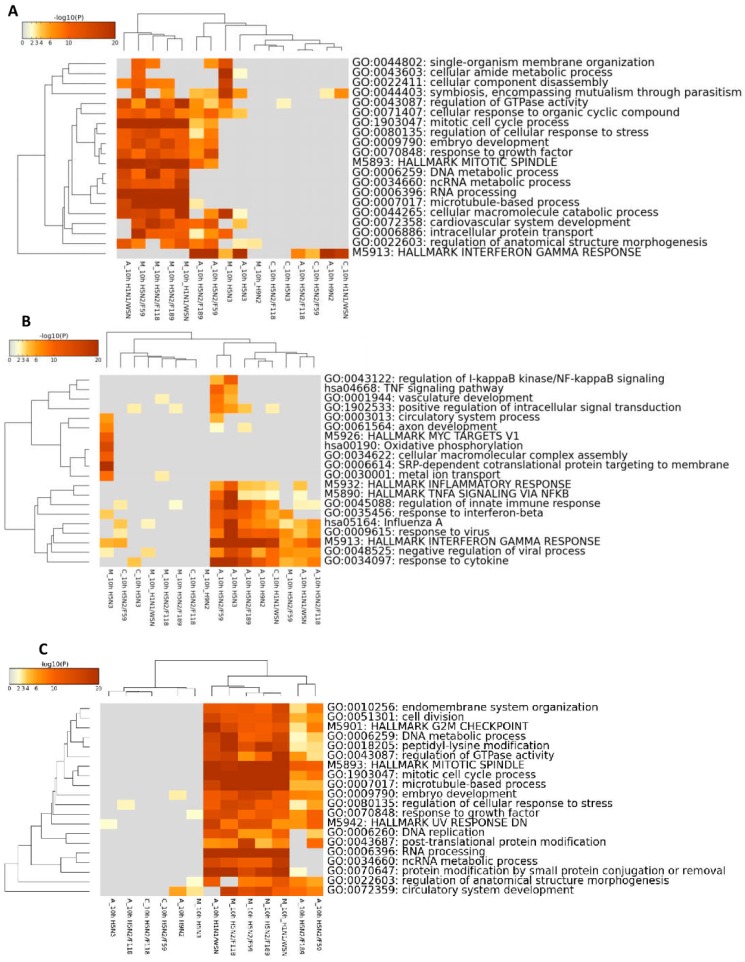
Functional annotation of the DEGs in A549, CEF and MDCK cell lines during influenza A virus (IAV) infections at 10 hpi. (**A**) Functional annotation of both up-and down-regulated genes together; (**B**) functional annotation of up-regulated genes; (**C**) functional annotation of down-regulated genes. The suffix labelling A, C and M indicates the cell type A549, CEF and MDCK respectively, (e.g., A_10h H1N1/WSN, A549 cells infection with H1N1/WSN virus at 10 hpi).

To compare the expression changes of the three cells side-by-side, we mapped the chicken and canine genes/probes together using human gene/probe orthologs as common basis (Table S1). As the majority of the expression changes were observed at 10 hpi, the DEGs at this time point of infections were used for further functional annotation and pathway enrichment analysis. A total of 10,845 genes were differentially expressed in at least one of the events at this time point. The functional annotations of the DEGs in each cell type and virus infections were described in Figure 2.

Generally, the functional annotations of the DEGs were related to cell cycle, cellular organization, metabolic processes and immune response pathways (Figure 2A). Detailed pathway enrichment analysis of the up-and down-regulated genes separately is given in Figure 2B,C, respectively. The up-regulated genes are associated with immune response (interferon and inflammatory) responses, intracellular signaling and cellular transport (Figure 2B). The interferon and inflammatory responses were generally up-regulated in A549 cells infected with all viruses and also for H1N1/WSN virus in CEF cells, and very slightly H5N2/F59 and H5N3 virus infections in MDCK cells (Figure 2B). On the other hand, IAV infection down-regulates genes associated with cell cycle and nucleic acid metabolism with strongest effect in MDCK cells, followed by A549 cells (with the H1N1/WSN, H5N2/F189, H5N2/F59 viruses) (Figure 2C). No such characteristics were observed in all infections of CEF cells, in A549 cells with H5N3, H5N2/F118, H9N2 virus infections and in MDCK cells with H5N3 virus.

Overall, the data suggests that IAV infections in A549 cells mainly induce interferon and inflammatory pathways, while virus infections also induce cell cycle pathway arrest in MDCK cells and variably in A549 cells in a strain dependent manner. Conversely, relatively very low interferon and no inflammatory responses were observed in CEF infections and pathways related to cell cycle were not arrested in CEF cells.

### 2.4. Correlation and Clustering of DEGs at 10 hpi

**Figure 3:**
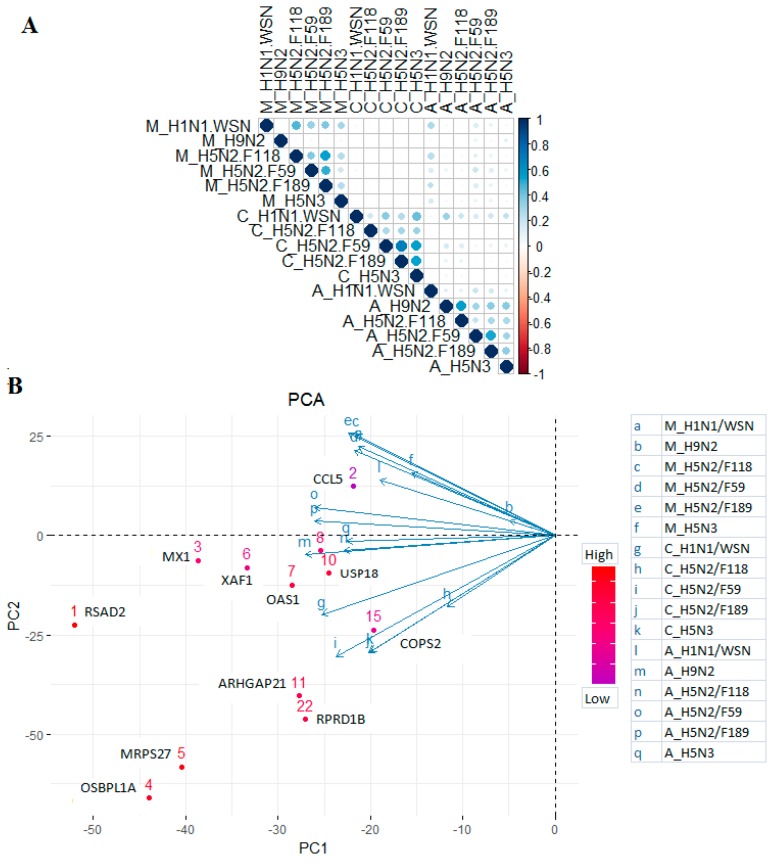
Correlation and clustering of the DEGs. (**A**) Correlation matrix of the DEGs in the three cell lines across the IAV infections at 10 hpi; (**B**) principal component analysis of the DEGs to identify the determinat genes in each cluster (species).

Correlation analysis of DEGs at 10 hpi (Figure 3A) indicated that the expression correlation of different viruses in one cell type is higher compared to the expression correlation of a single virus in different cell types. For instance, the expression of DEGs in MDCK cells following H1N1/WSN virus infection at 10 hpi (M_H1N1.WSN) was correlated better with the other viruses infecting the same cell type (M_H5N2.F118, M_H5N2.F59, M_H5N2.F189 and M_H5N3 viruses) compared to the same virus infecting A549 cells (A_H1N.WSN) and CEF cells (C_H1N1.WSN) (Figure 3A). This phenomenon is also similar for other viruses (Figure 3A). In addition, principal component analysis (PCA) clustered the expression patterns at 10 hpi into three groups (Figure 3B) that clustered mainly towards the cell type rather than IAV strains. DEGs associated with expression in CEF cells (Principal component 1 (PC1)) include *OSBPL1A*, *MRPS27*, *RPRD1B*, *ARHGAP21* and *COPS2*, while *CCL5* expression was associated with MDCK cells (Figure 3B). Interferon stimulatory genes (ISGs) expressions are mainly associated with A549 (e.g., *RSAD2*, *OAS1*, *USP18*, *XAF1*, *MAX1*, *SAMD9L*) followed by CEF cells (Figure 3B). Consequently, the above listed genes comprise the majority of cell type-specific gene expression differences.

**Figure 4:**
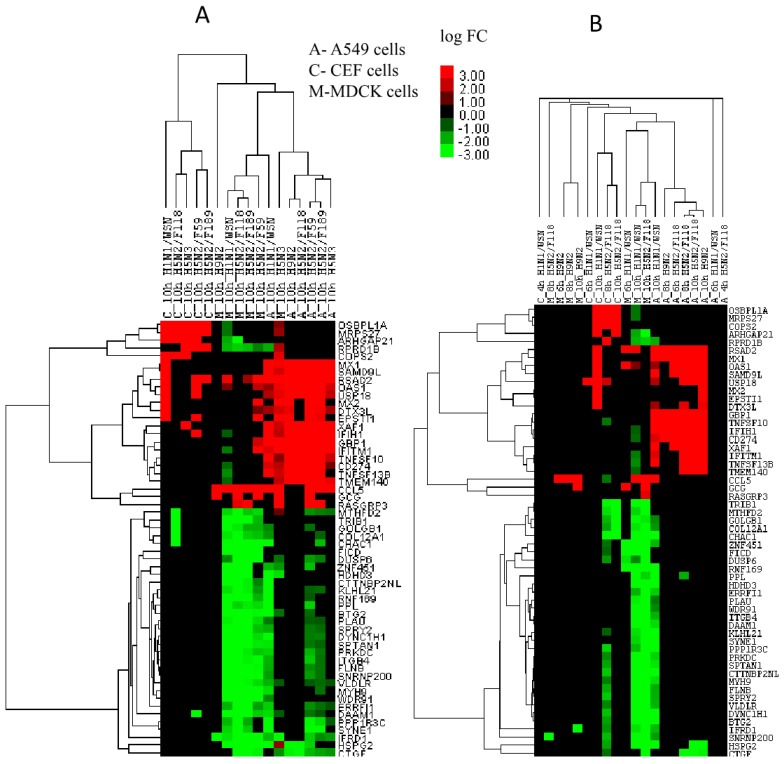
Hierarchical clustering of the top 56 DEGs across the cell lines and virus infections. (**A**) hierarchical clustering of the top 56 DEGs at 10 hpi; (**B**) hierarchical clustering of the top 56 DEGs during temporal infections in three viruses (H1N1/WSN, H5N2/F118 and H9N2).

The hierarchical clustering of the top 56 differentially expressed genes (using Gene cluster 3.0) is in accordance with the PCA analysis that expression patterns of the same cells were clustered together except in M_H5N3 virus (in MDCK) and A_H1N1/WSN virus (in A549 cells) at 10 hpi (Figure 4A).

The temporal expression of these genes in three of the viruses (H1N1/WSN, H5N2/F118 and H9N2) in the three cell types indicated few changes before 10 hpi. Some of the interferon-stimulated genes (ISGs) were up-regulated at 6 and 8 hpi during the avian virus infection in A549 cells. In MDCK cells, H1N1/WSN virus induces *RSAD2* and *OAS1*, while H9N2 virus induces *CCL5* from 6 hpi onwards (Figure 4B). Taken together, this data suggested expression signature differences in species during IAV infections also in terms of details of the antiviral response.

### 2.5. DEGs Associated with Metabolic Pathways

**Figure 5:**
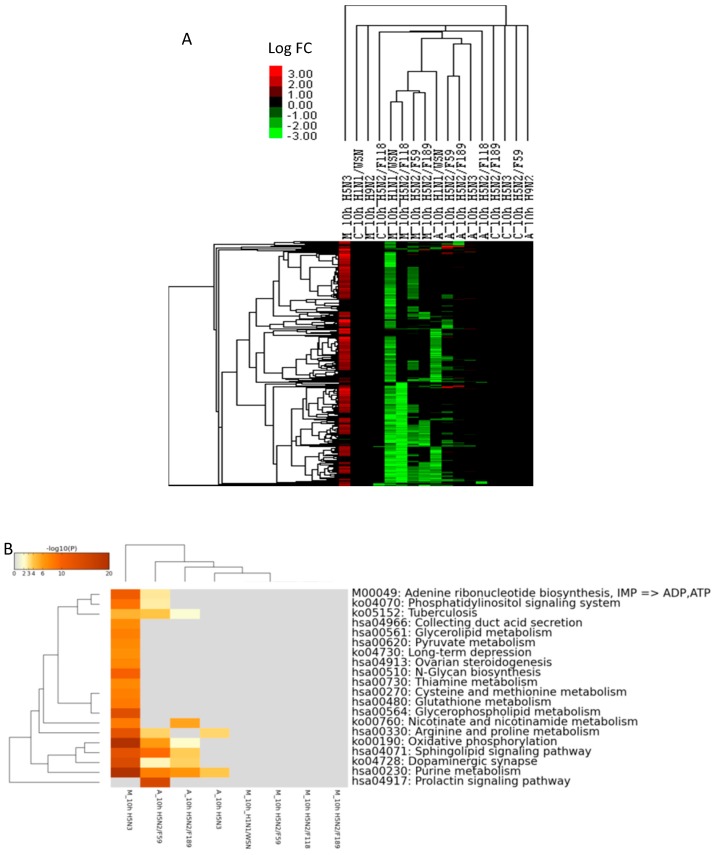

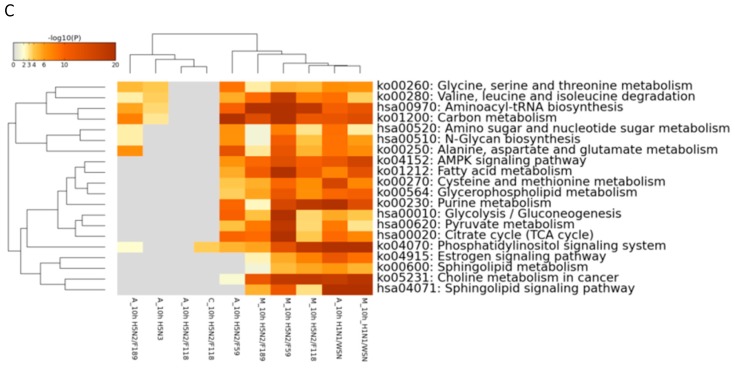
DEGs and pathways involved in metabolic pathways. (**A**) Hierarchical clustering and heat map of the DEGs involved in metabolic pathways; (**B**) top 20 up-regulated metabolic pathways; (**C**) top 20 down-regulated metabolic pathways.

Detailed investigation of DEGs involved in metabolic pathways identified 665 genes differentially expressed at least once across the three cell types and the virus infections at 10 hpi. The hierarchical clustering and heat map of the 665 DEGs was described in Figure 5A. As indicated in Figure 5A, most of the metabolic pathways were up-regulated during H5N3 virus infections in MDCK cells. Conversely, many of the genes were down-regulated in MDCK cells (H1N1/WSN and H5N2 virus infections), and A549 cells (H1N1/WSN and H5N2/F59 virus infections). Almost no effects on metabolic genes were observed in CEF cells infected with all of the tested viruses. Similarly, H9N2 virus was silent in changing the metabolic state of the three cell types.

For pathway enrichment analysis, we identified the up- and down-regulated genes in each cell and virus infection setup. The KEGG pathway analysis of the up-regulated metabolic genes indicated that energy (oxidative phosphorylation), lipid, amino acid, nucleic acid, glycan and vitamin metabolic pathways were enriched in H5N3 virus infections in MDCK cells at 10 hpi (Figure 5B). No induction of the metabolic pathways was found in MDCK cells in other virus infections. To the reverse, nucleic acid, amino acid, lipid, carbohydrate and glycan metabolic pathways were down-regulated in MDCK cells (in H1N1/WSN, and H5N2 infections) and A549 cells (in H1N1/WSN and H5N2/F59 virus infections) (Figure 5C).

## 3. Discussion

There are several factors that can influence the capacity of avian and mammalian viruses to replicate in different cell types. One current paradigm is that species adaptation involves the interactions of multiple virus proteins with specific cellular proteins, and we can hypothesis that these interactions induce changes in cell transcriptome. These in turn would be expected to induce changes in the expression and activity of a myriad of other cellular pathways that play a diverse role, from the host response to infection to virus-induced changes in cell metabolism. At the very basic level these virus-induced changes could determine the capacity of the virus to productively replicate in these cell types. However, although these host cell proteins perform similar activities in these cells, it is likely that the sequences of the same proteins in the avian or human background will differ. This would be expected to influence the interaction of the virus proteins, and in turn these differences may lead to fundamental changes in the host expression profile. Such changes in gene expression would be expected to have a major impact on virus replication and transmission.

Combining gene expression results from multiple species of a specific condition (e.g., disease) can lead to additional findings related to cross-species conservation or host-specificity that cannot be seen in a single species result alone [13]. However, cross-species comparison of microarray data is complicated because of several factors including platform variations, probe quality, laboratory effects, dynamic environment, genetic background and organism annotation status [13]. Similar tissues have significant conserved expression patterns across species [14]. Comparison of host-gene expression signatures between species is important to evaluate the host-specific changes during IAV infections. Side-by-side comparison of single genes expression in different species could be possible by mapping orthologous genes of the species. This approach could also evaluate whether the experiment in one species is representative of the others. To study these effects, we performed a comprehensive human orthologs mapping of chicken and canine genes to compare microarray results from infections in the three cell types under identical experimental condition and with the same influenza virus isolates. This therefore allowed us to compare the expression profile of homologous proteins in these different cell types, through mapping of their genes to human orthologs.

In this investigation, we identified highly expressed gene signatures that could partially explain the role of host factors in host adaptation of IAVs. Generally, regardless of the IAV subtype infection, the key difference between mammalian and avian (chicken) cell line expression signatures come from vesicular transport, apoptosis and interferon responses. We have selected highly differentially expressed genes identified as being characteristic for the different signatures in the principal component analysis to explain this phenomenon. *OSBPL1A*, *MRPS27*, *COPS2*, *ARHGAP21* were highly expressed in CEF cells (Figure 4). *OSBPL1A* and *ARHGAP21* are involved in vesicular trafficking while *MRPS27*, *COPS2* have an anti-apoptotic role. Conversely, *CCL5* which is highly expressed in MDCK and A549 cells may induce apoptosis and together with *CCL5*, ISGs and pre-apoptotic signals (*XAF1*) would induce apoptosis and hinder the replication of IAV in A549 cells.

Oxysterol binding proteins (*OSBPs*) are involved in cell signaling, cytoskeletal organization, lipid homeostasis, sphingolipid (SM) metabolism and *LXR/ABCA1* regulations [15]. OSBP-related protein (ORP) 1 is one of 12 proteins in the family. It has two variants *ORP1S (OSBPL1A)* and *ORP1L (OSBPL1B)* mainly localized in the cytosol and late endosome respectively [15]. *ORP1S* binds both cholesterol and oxysterols with high affinity and this observation suggested that sterol binding promotes *ORP1S* movement from the cytoplasm to the nucleoplasm [16]. Overexpression of *ORP1S* enhanced sterol transport from the plasma membrane (PM) to the endoplasmic reticulum (ER) and lipid droplets (LDs) in HeLa cells [17], which can be used for new membrane synthesis. It has been observed that depletion of membrane cholesterol from the IAV envelope significantly reduced the IAV infectivity in MDBK [18] and MDCK cells [19]. Conversely, virus infectivity was maintained upon addition of exogenous cholesterol [18,19]. It was also suggested that IAV virus genome segments are transported to the plasma membrane by cholesterol-enriched recycling endosomes [20].

*ORP1L*, a variant of *OSBPL1A*, co-localizes and interacts with GTP-bound *RAB7*. *ORP1L* modifies the functional cycle of *RAB7* and interferes with late endosome (LE)/lysosome endocytic membrane trafficking [15]. Cholesterol levels in LE are sensed by *ORP1L* and depending on the concentration of cholesterol *ORP1L* changes its conformation and guides the transport of LE to the minus and plus end of microtubules accordingly [21]. *ORP1L* forms *RILP-RAB7-ORP1L* complex to recruit dynein motors which initiate translocation of LE to the microtubule minus end [22]. Overexpression of *ORP1L* has been shown to induce protein clusters in endosomes in the perinuclear area of the cell [23]. This suggests that it might also have a role in the transport of IAV containing LE to the nucleoplasm.

It has been demonstrated that, *RAB5* and *RAB7* are required for IAV (H1N1/WSN and H3N2) infection [24] and *RAB7* was mainly involved in the intermittent and confined movement of the virus in the perinuclear region [25]. *RAB7* is LE/lysosomal small GTPase that is required for the fusion of many PH-dependent viruses, including IAV. The known antiviral *IFITM3* partially co-localizes with *RAB7* and *LAMP1*, and overexpression of *IFITM3* by IFN treatment expands these components [26]. Recently, it was demonstrated that *IFITM3* disrupts intracellular cholesterol homeostasis to block viral entry [27] through interaction with VAPA and preventing its association with *OSBP*. By altering *VAPA-OSBP* function, *IFITM3* induces a marked accumulation of cholesterol in multi-vesicular bodies and late endosomes, which inhibits the fusion of intraluminal viron-containing vesicles with endosomal membranes and thereby blocks release into the cytosol [27]. Further, the expression patterns of interferon-induced transmembrane protein (*IFITM)* genes showed little response in chicken compared to higher expressions in duck [28]. Duck *IFITM3* also mediates restriction of H1N1, H6N2 and H11N9 virus infections [29]. In our investigation, *IFITIM3* was not differentially regulated in CEF cells, while it was up-regulated in MDCK (H5N2/F59, H5N3) and A549 (H5N2/F59 and H5N2/F189) cells. Taken together, increased expression of *OSBPL1A* in CEF cells could contribute to increased rate of virus replication compared to MDCK and A549 cells [10]. The highly expressed *ARHGAP21* in CEF cell infections at 10 hpi, was also reported to be involved in trafficking through the control of CDC42 activity [30]. The overexpression of *ARHGAP21* negatively regulates the transport of IAV neuraminidase (NA) to the cell surface and inhibits IAV (H1N1/WSN) virus replication in A549 cells [31].

Mammalian mitochondrial ribosomal protein of small subunit 27 (*MRPS27*) is one of the pentatricopeptide repeat domain (PPR) proteins required for mitochondrial protein synthesis (translation). Its knockdown is associated with low level of respiratory complexes and cytochrome c oxidase activity (*COXI*, *COXII*) [32], and disruption of mitochondrial membrane potential (MMP) [33]. Viruses control various cellular organelles for efficient replication and to escape from host defense and immune responses. In addition to its bioenergetics activity, mitochondria are implicated in antiviral response and apoptosis [34]. Apoptosis is associated with decreased mitochondrial inner membrane potential, increased cytochrome c release; a reduction in cytochrome c oxidase activity; caspase activation and DNA damage [35]. Pathological cell death induced by ischemia/reperfusion, intoxication with xenobiotics, neurodegenerative diseases, or viral infection also relies on MMP as a critical event [36].

*COPS2* is one of the *COP9* signalosome proteins localized in both cytoplasm and nucleus [37,38]. It is implicated in the pluripotent maintenance of human and mouse embryonic stem cells (ESCs), somatic cell reprogramming, and regulation of ubiquitin-dependent proteasomal degradation [38,39]. Knock down of *COPS2* resulted in decreased proliferation rate and G2M arrest in mouse ESCs, while its overexpression increased somatic cell reprogramming in virus (retrovirus) expressing cells [38]. In our investigation, we have observed down-regulation of genes associated with cell proliferation including the G2M checkpoint (Figure 2C) in MDCK and A549 cell infections, but not in CEF cells. This suggests that the overexpression of *COPS2* could have a role in maintaining CEF cell proliferation or reprogramming IAV infected cells. However, the overexpression of *COPS2* in CEF cells is not consistent in infection with all IAV subtypes.

In this study we also investigated the effect of virus infection on DEGs that are involved in metabolic pathways. We noted that the H5N3 virus induced oxidative phosphorylation, nucleic acid, amino acid, lipid and glycan metabolisms in MDCK cell infections. To evaluate whether these metabolic pathways were pro-viral or antiviral, we used 194 genes that were previously identified as being involved in metabolic pathways from pro-viral siRNA screen genes [40] (Table S2). We compared the metabolic pathways from the pro-viral siRNA screen with the DEGs indentified in the our analysis following virus infection. From the siRNA screens metabolic pathways, oxidative phosphorylation and other metabolic pathways were relevant for the replication of IAVs (Figure S1a). Induction (up-regulation) of these pathways particularly in H5N3 virus could play important role for its increased replication (Figure S1a). In addition, purine metabolism was also induced by H5N2/F59, H5N2/F118 and H5N3 virus infections in A549 cells (Figure S1a). Interestingly, these pathways important for replication were down-regulated in MDCK cells (when infected with H1N1/WSN and H5N2 viruses) and A549 cells (when infected with H1N1/WSN and H5N2/F59 viruses) (Figure S1b). Very few down-regulated metabolic pathways were observed in A549 cells infected with H5N2/F189 virus. Overall, the results of the transcriptomic analysis suggested that induction or maintaining the metabolic pathways of the cells during IAV infections could be important for replication of IAVs in a virus- and cell-type specific manner. This is consistent with previous reports indicating that glycolysis, lipid and cholesterol biosynthesis were increased in H1N1/PR8 infected MDCK cells [41,42]. Similarly, a proteomic study in pH1N1 infected NHBE cells showed increased level of proteins associated with oxidative phosphorylation, fatty acid metabolism, glycolysis, TAC-cycle, pentose phosphate pathways. In addition, the induction of the metabolic pathways was associated with increased survival of the virus infected cells [43]. It was suggested that activation of cellular metabolism in IAV infections was a compensatory mechanism of the cell for resources (energy, lipid, amino acids and nucleic acids) which were depleted during virion production [43]. Recently, Meyer et al., 2017 demonstrated increased expression of mitochondrial genes associated with oxidative phosphorylation in LPAI H6N2 and H7N1 viruses in a chicken lung epithelial cell line (CLEC213) [44]. The same study showed that inhibition of oxidative phosphorylation using oligomycin reduced virus replication without cell toxicity [44]. Overall, the present data suggested that induction of or maintaining specific metabolic pathways during IAV infections could be important for cell survival and IAV replication.

## 4. Materials and Methods

### 4.1. Cell Culture and Viruses

Human alveolar basal epithelial (A549, ECACC 86,012,804) and Madin-Darby canine kidney (MDCK, ECACC 84,121,903) were obtained from European Collection of Cell Cultures and were maintained in Dulbecco’s Modified Eagle’s medium (DMEM) (Invitrogen, Carlsbad, CA, USA) with 10% FBS and 1% penicillin/streptomycin (pen/strep) (Invitrogen). Chick embryo fibroblasts (CEF) were prepared from 8 to 10 day-old chick embryos and maintained in DMEM containing 10% FBS and pen/strep. The LPAI isolates A/Duck/Malaysia/F118/2004 (H5N2/F118), A/Duck/Malaysia/F189/2004 (H5N2/F189), A/Duck/Malaysia/F59/2004, A/Duck/Singapore-Q/F119/1997 (H5N3) and A/Duck/Malaysia/02/2001 (H9N2) were obtained from the Agri-Food and Veterinary Authority of Singapore and have been described previously [9]. The laboratory-adapted A/WSN/1933 (H1N1/WSN) (VR-1520) was purchased from American Type Culture Collection (ATCC, Manassas, VA, USA). All virus stocks were prepared in 9 to 11-day-old embryonated chicken eggs, and the infectivity assessed using standard overlay plaque assay or by determining the TCID_50_ in MDCK cells. Virus infections in A549, MDCK and CEFs were carried out in Dulbecco’s Modified Eagle’s medium (DMEM) (Invitrogen) with 2% FBS and pen/strep at 37 °C in 5% CO_2_. Virus was allowed to absorb to the cell monolayer for 1 h at 37 °C, after which it was removed and replaced with prewarmed DMEM (with 2% FCS with pen/strep).

### 4.2. Microarray Data Analysis and Functional Annotations

The MDCK, A549 and CEF monolayers were either mock-infected or virus-infected at an multiplicity of infection (MOI) = 4 as described previously [10]. Briefly, the total RNA was extracted from approximately 1 × 10^7^ cells using the RNeasy MiniKit (Qiagen, Hilden, Germany) and double-stranded cDNA was synthesized from 3 μg of total RNA with the GeneChip One-Cycle cDNA synthesis kit (Affymetrix, Santa Clara, CA, USA), followed by synthesis of biotin-labelled cRNA using the GeneChip IVT labelling kit (Affymetrix). After cRNA fragmentation, the biotin-labelled cRNA was hybridized to the GeneChip Canine Genome 2.0 Array (Affymetrix), the GeneChip Chicken Genome Array (Affymetrix) and Genechip Human Genome HG U133 Plus 2.0 Array (Affymetrix) as appropriate to the host cell line being analyzed. The arrays were washed and stained using the Hybridization, Wash and Stain Kit (Affymetrix) and the GeneChip Fluidic Station 450 (Affymetrix) according to the standard Affymetrix protocols. Finally, the arrays were scanned with the GeneChip scanner 3000 (Affymetrix).

Affymetrix .CEL files were generated from GeneChip Operating Software (GCOS) version 5.0 and GEO2R (limma, R-package) was used to analyze the microarray data. Differentially expressed probe sets were selected with statistical significant change, the Benjamini-Hochberg False Discovery method (adjusted *p*-value) <0.05) and |Log Fold change (FC)| > 1 (changes in virus infection in respect to corresponding mock). A single gene might be represented by more than one probes, hence those genes with adjusted *p*-value < 0.05 and their probe mean |log FC| > 1 were considered as differentially expressed genes (DEGs). Customized Perl script was used to summarize the genes (removing the redundancies) and probe sets with highest |log FC| > 1 were used to construct heat maps.

The top 56 DEGs were selected based on their mean expression values across all the viruses at 10 hpi and these selected genes were clustered (unsupervised hierarchical clustering) using Gene Cluster version 3 [45]. Functional annotations (Pathway and biological process) of the DEGs were analyzed using Gene Annotation and Analysis Resource-Metascape (http://metascape.org/gp/index.html#/main/step1) [46] and *q*-value < 0.01 was used to select the top 20 statistically significant pathways. All microarray data were deposited in NCBI Gene Expression Omnibus (GEO) DataSets with accession number GSE31524.

### 4.3. Statistical Analysis

Descriptive statistics, chi-square test with 95% confidence interval (CI), Fisher’s exact test (for values lower than 5), were analyzed using STATA version 11 to determine the difference in the number of DEGs among the three cell lines in the six IAV infections at 5 time points. Cross-correlation matrix and principal component analysis were analyzed using R program R commander package [47] and custom Perl script was used to refine and count probes and genes.

### 4.4. Annotation of Affymetrix Probes

The overall overview of the materials and methods was described in Figure 1. Probe annotation information for Chicken Genome array, Canine_2 Genome array and Human 133A 2 plus were obtained from AffymetrixNetaffxTM website (document deposition) [48].The non-annotated probes with no Ensembl ID in chicken and canine chips were isolated and their corresponding sequences were retrieved from chicken probe sequences, FASTA (8MB 08/20/08) [49] and Canine_2 probe sequences (FASTA, 9MB) [50] files from Affymetrix. Whole genome sequences of chicken (*Gallus gallus* (17,108 genes) and canine (*Canis familiaris* (24,580 genes) with their corresponding Ensembl IDs were downloaded from Ensembl (Ensemble genes 83) http://dec2015.archive.ensembl.org/index.html [51]. The probe sequences with no ensembl IDs in the chicken and canine Affymetrix chips were aligned against their corresponding whole genome gene sequences from ensembl, using ncbi-blast+ package [52]. A single probe sequence can align with more than one gene; hence, aligned genes with lowest *p*-value, higher sequence % identity and higher frequency were identified as the best hits for annotation.

### 4.5. Mapping Human Orthologs of Chicken and Canine Genes/Probes

Human orthologs information for chicken and canine were obtained (1) from Affymetrix web document depository (2) by analyzing orthologs using “Orthologous Matrix” (OMA) database [12]. Orthologs between two species (*Gallus gallus* vs. *Homo sapiens*) and (*Canis familiaris* vs. *Homo sapiens*) were analyzed using OMA, and the results were obtained with ensembl gene IDs. The chicken and canine probes orthologs to Human Genome HG U133 plus 2.0 Array were mapped to ensembl IDs. The OMA orthologs genes were also mapped to corresponding Affymetrix probes using the Affymetrix and our annotations. The orthologs from the two datasets were combined. Chicken vs. human and canine vs. human, and human orthologs of both canine and chicken were used for further analysis.

## 5. Conclusions

In conclusion, re-annotation of the chicken and canine Affymetrix chips through human ortholog mapping allowed us to find more differentially expressed genes that can be mapped to functional pathways in our experimental design using multiple different IAV isolates. The number of orthologous genes whose expression changed when infected with the IAVs used in this study were in the order of MDCK cells > A549 cells > CEF cells. We have identified important host species signatures that could help the replication of IAV while not affecting the overall cellular physiology (e.g., apoptosis and metabolic pathways). In future studies we will validate the role that these genes play during virus replication e.g., using knock in/out experiments in the three cell types. In addition, the functional relevance of these genes to IAV infection will be assessed using primary cell culture, which will allow us to assess their relevance during IAV host range adaptation.

## Figures and Tables

**Table 1:** Annotation of affymetrix probe sets with no ensembl identifications (IDs).

Probe Annotation	Chicken Genome Array	Canine Genome 2.0 Array
Total number of affymetrix probes	38,535	43,035
Total number of genes (ensembl IDs)	12,639	16,602
Probes without ensembl ID annotation	16,055	13,195
Probes annotated in this study (new)	15,979	13,162
Genes corresponding to the new annotation	7412	6869
New annotated genes represented by other affymetrix probes	6229	5899
New annotated genes unique to this study	1183	970
Total number of fully annotated probes	38,474	43,006
Total number of fully annotated genes	14,007	17,813

The total number of genes (from ensembl genes 83) for Chicken and Canine are 17,108 and 24,580 respectively.

**Table 2:** Human ortholog mapping of *Canis familiaris* (Canine) and *Gallus gallus* (Chicken) genes/probes.

Ortholog Mapping	Gene/Probe	Chicken Orthologs	Canine Orthologs
Affymetrix orthologs	Probes	14,415 (37.04)	15,053 (34.97)
	Genes	8654 (61.78)	9109 (51.13)
Orthologous Matrix (OMA) orthologs	Genes	11,674 (83.34)	16,002 (89.83)
OMA orthologs common to the full annotation (ensemble ID)	Genes	11,220 (80)	15,284 (85.8)
Mapping OMA orthologs to Affymetrix probes	Genes	11,175 (79.78)	15,552 (87.30)
	Probes	32,244 (83.67)	38,755 (90.05)
Total Orthologs (OMA + Affymetrix)	Genes	11,788 (84.15)	15,577 (87.44)
	Probes	34,335 (89.10)	39,436 (91.63)

**Table 3:** Comparison of orthologous differentially expressed genes (DEGs) in influenza A virus (IAV) infected A549 and Madin-Darby canine kidney (MDCK) cells.

Virus Isolates	Post Infection	A549	MDCK	Common	*p*-Value
All DEGs					
In all isolates	over all time points	5684	13,737	5301	<0.001
H1N1/WSN	6 h	2	135	0	1 *
	10 h	4173	10,683	3619	<0.001
H9N2	6 h	0	2	0	-
	8 h	19	1	0	1 *
	10 h	70	11	2	0.001 *
H5N2/F118	4 h	1	0	0	-
	6 h	33	0	0	-
	8 h	83	17	1	0.087 *
	10 h	93	6106	40	0.45
H5N2/F59	10 h	2269	2664	729	<0.001
H5N2/F189	10 h	974	2194	281	<0.001
H5N3	10 h	281	9691	192	0.033
Up-regulated genes					
In all isolates		1282	9765	871	<0.001
H1N1/WSN	6 h	2	93	0	1 *
	10 h	145	79	11	<0.001
H9N2	6 h	0	2	0	-
	8 h	18	1	0	1 *
	10h	61	8	2	<0.001
H5N2/F118	4 h	1	0	0	-
	6 h	30	0	0	-
	8 h	54	3	1	0.01
	10 h	40	131	2	0.044 *
H5N2/F59	10 h	957	83	35	<0.001
H5N2/F189	10 h	459	31	6	<0.001
H5N3	10 h	189	9664	136	<0.001
Down-regulated genes					
In all isolates		4675	11,762	4220	<0.001
H1N1/WSN	6 h	0	42	0	-
	10 h	4028	10,604	3532	<0.001
H9N2	6 h	0	0	0	-
	8 h	2	0	0	
	10 h	9	3	0	1 *
H5N2/F118	4 h	0	0	0	-
	6 h	3	0	0	-
	8 h	29	14	0	1 *
	10 h	53	5975	28	0.03
H5N2/F59	10 h	1312	2581	587	<0.001
H5N2/F189	10 h	515	2163	213	<0.001
H5N3	10 h	92	27	5	<0.001

* Fisher’s exact test, - not available.

**Table 4:** Comparison of ortholog DEGs in IAV infected A549 and Chick embryo fibroblasts (CEF) cells.

Virus Isolates	Post Infection	A549	CEF	Common	*p*-Value
All DEGs					
In all isolates	over all time points	4743	3716	2099	<0.001
H1N1/WSN	4 h	0	1	0	
	6 h	1	14	0	1 *
	10 h	3541	45	13	0.866
H9N2	8 h	11	0	0	
	10 h	42	0	0	
H5N2/F118	6 h	18	0	0	
	8 h	51	3645	18	0.498
	10 h	65	84	0	1 *
H5N2/F59	10 h	1894	19	7	0.014
H5N2/F189	10 h	786	5	1	0.292 *
H5N3	10 h	219	18	1	0.287 *
Up-regulated					
In all isolates		980	159	32	<0.001
H1N1/WSN	6 h	1	8	0	
	10 h	91	42	7	<0.001
H9N2	8 h	12	0	0	
	10 h	31	0	0	
H5N2/F118	6 h	18	0	0	
	8 h	27	103	2	0.023 *
	10 h	24	11	0	1 *
H5N2/F59	10 h	756	13	5	0.001 *
H5N2/F189	10 h	328	5	1	0.132 *
H5N3	10 h	136	18	1	0.189 *
Down-regulated					
In all isolates		3983	3573	1870	<0.001
H1N1/WSN	4 h	0	1	0	
	6 h	0	6	0	
	10 h	3450	3	1	1 *
H9N2	8 h	1	0	0	
	10 h	11	0	0	
H5N2/F118	6 h	0	0	0	
	8 h	24	3542	9	0.425
	10 h	41	73	0	1 *
H5N2/F59	10 h	1138	6	2	0.107 *
H5N2/F189	10 h	458	0	0	
H5N3	10 h	83	0	0	

* Fisher’s exact test, - not available.

## Supplementary Materials

Supplementary materials can be found at www.mdpi.com/1422-0067/18/11/2295/s1.
