# Supplementary material for: Inter-Species Host Gene Expression Differences in Response to Human and Avian Influenza A Virus Strains
Source: Int J Mol Sci. 2017 Nov 1;18(11):2295. doi: 10.3390/ijms18112295 (PMC5713265; doi:10.3390/ijms18112295)
Supplement: Supplementary file 1 [file ijms-18-02295-s001.zip › Supplementary Files/Figure S1.pdf]

a)

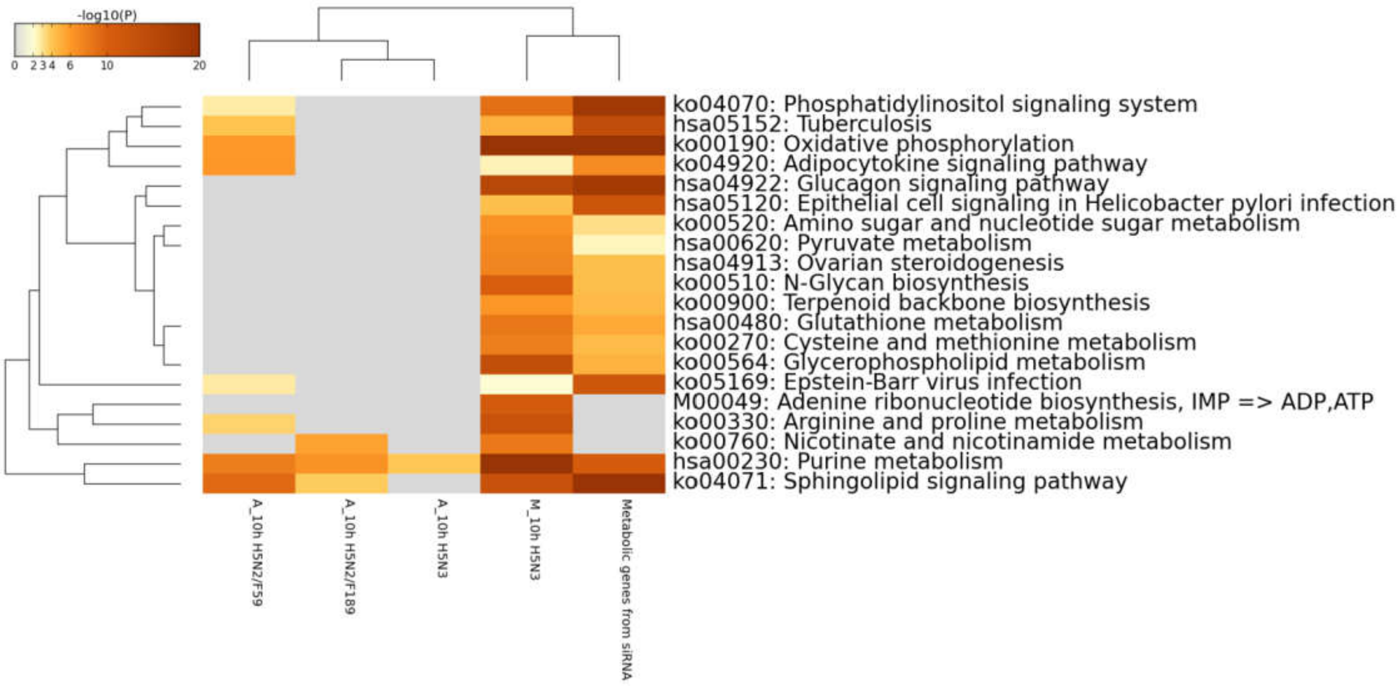

b)

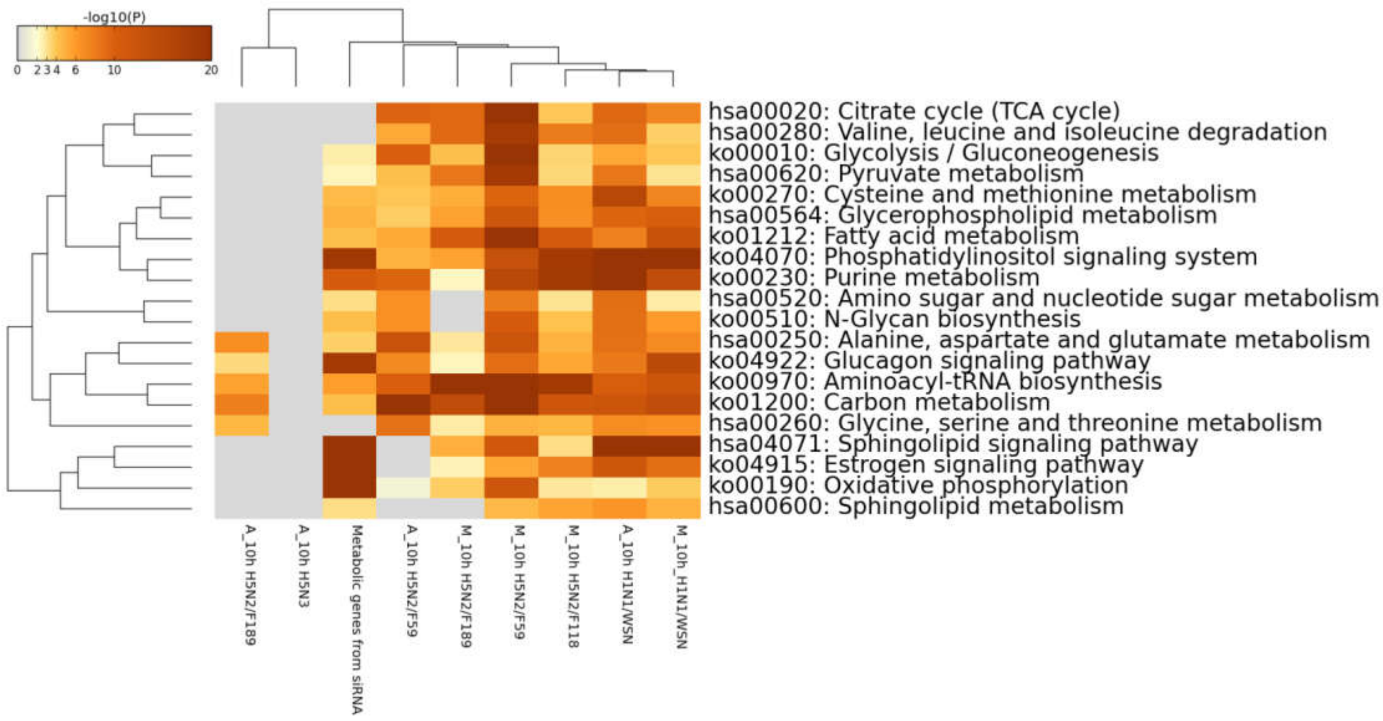

Figure S1: Comparison of metabolic pathways between the pro-viral siRNA screens and DEGs. (**a**) Top 20 pro-viral metabolic pathways (from siRNA screens) across the up-regulated metabolic pathways across the cells and the virus infections; (**b**) Top 20 pro-viral metabolic pathways (from the siRNA screens) across the down-regulated metabolic pathways across the cells and the virus infections; Figure captions should move under supplementary figure.
